# A Study of Peripheral Blood Parameters to Predict Response to Induction Chemotherapy and Overall Survival in Advanced Laryngeal Squamous Cell Carcinoma

**DOI:** 10.3390/curroncol29090509

**Published:** 2022-09-09

**Authors:** Jiaqi Xu, Yifan Yang, Qi Zhong, Lizhen Hou, Hongzhi Ma, Yang Zhang, Ling Feng, Shizhi He, Meng Lian, Jugao Fang, Ru Wang

**Affiliations:** Department of Otorhinolaryngology Head and Neck Surgery, Beijing Tongren Hospital, Capital Medical University, Beijing 100730, China

**Keywords:** larynx, carcinoma, peripheral blood parameters, nomogram, induction chemotherapy

## Abstract

Purpose: the purpose of this study was to screen peripheral blood parameters and construct models predicting the prognosis and induction chemotherapy (IC) response in locally advanced laryngeal squamous cell carcinoma (LSCC) patients. Methods: A total of 128 stage III/IVa LSCC patients (who required a total laryngectomy) were enrolled in a retrospective study from January 2013 to September 2020 at Beijing Tongren Hospital of Capital Medical University. Among them, 62 patients received IC (IC group), and 66 patients immediately underwent a total laryngectomy (TL) after diagnosis (surgery group). Demographic information and peripheral blood parameters were collected for further analysis. The overall survival (OS), progression-free survival (PFS), and disease-specific survival (DSS) were compared between the two groups. The prognosis and survival were also compared between patients with laryngeal function preservation (LFP) and those with TL. Results: The Receiver Operating Characteristic (ROC) curve for IC response in the IC group showed that the AUC of the blood model based on the four peripheral blood parameters of fibrinogen (FIB), platelet (PLT), high-density lipoprotein cholesterol (HDL), and albumin (ALB) was significantly higher than the TNM stage model’s AUC (0.7932 vs. 0.6568). We constructed a nomogram blood model to predict IC response (C-Index = 0.793). Regarding the OS of all patients, an ROC analysis for overall survival, the Kaplan–Meier (K-M) method with a log-rank test, and multivariate analysis indicated age, clinical stage, FIB, and hemoglobin (HGB) were independent prognostic factors for the OS of LSCC patients. The blood–clinical logistic model (AUC = 0.7979) was constructed based on the four prognosis factors, which were superior to the blood (AUC = 0.6867) or clinical models (AUC = 0.7145) alone to predict OS. We constructed a nomogram model based on age, clinical stage, FIB, and HGB to predict OS for LSCC patients (C-Index = 0.792). Besides this, there were no significant differences in OS, PFS, and DSS between IC and surgery groups or LFP and TL groups. Conclusion: Peripheral blood parameters help predict IC response and overall survival. Furthermore, induction chemotherapy significantly improves laryngeal function preservation without lowering the survival prognosis.

## 1. Introduction

Laryngeal squamous cell carcinoma (LSCC) is one of the most common head and neck carcinomas [1]. Despite diversified treatments, including radiotherapy, chemotherapy, biological therapy, and surgery, the 5-year overall survival remains 66% in the United States [2], 50–70% in Europe [3], and less than 50% in China [4]. In advanced LSCC patients, especially locally advanced T3T4a patients, a total laryngectomy (TL), which reduces quality of life, is often needed when surgery is chosen as the treatment modality [5]. Induction chemotherapy (IC) administrated before surgery or radiotherapy is an option to obtain information about radiosensitivity and choose the more appropriate treatment modality between surgery and radiotherapy [6]. However, IC responses are heterogeneous in patients, and some patients do not benefit from it [7]. Therefore, it is necessary to find biomarkers predicting IC response and prognosis in advanced LSCC patients [8].

Peripheral blood parameters in blood routine tests, biochemical examination, and coagulation tests have been used to predict the prognosis of LSCC patients with ease of testing [9]. However, they still lack reliable predictive factors for IC response and models for survival [10].

Our research screened peripheral blood parameters and conducted models to predict prognosis and IC response in advanced LSCC patients, providing some references for comprehensive treatment choice.

## 2. Materials and Methods

### 2.1. Patients

**Figure 1:**
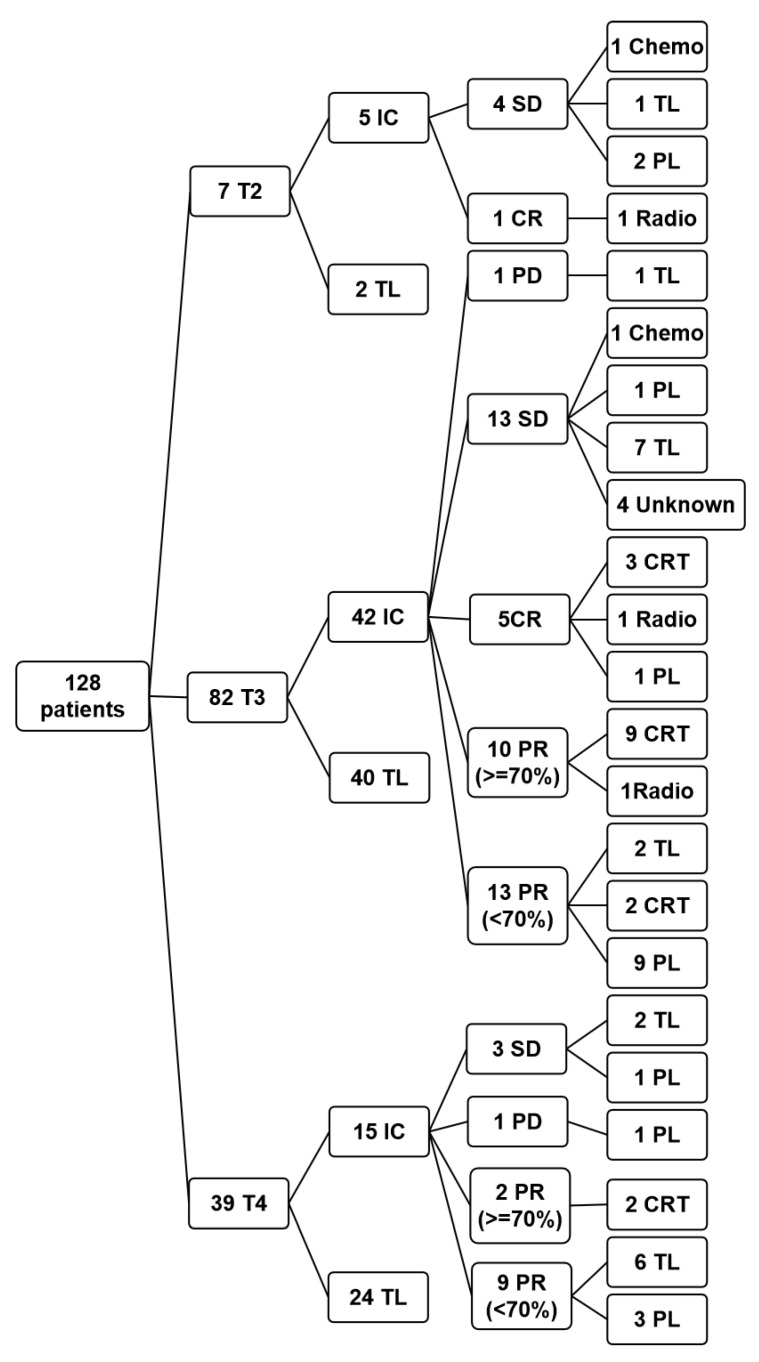
A flow diagram about the treatment protocol and groups. IC: induction chemotherapy; TL: total laryngectomy; SD: stable disease; PD: progressive disease; PR: partial response; CR: complete response; Chemo: chemotherapy; Radio: radiotherapy; PL: partial laryngectomy; CRT: current radiochemotherapy; Unknown: these patients preferred not to provide information about their treatments.

We enrolled 128 stage III/IVa (according to the 2017 American Joint Committee on Cancer Staging Classification) LSCC patients (who required a total laryngectomy) for our retrospective research from January 2013 to September 2020 at Beijing Tongren Hospital of Capital Medical University (CMU) [11]. All patients had complete medical records, including treatment responses and follow-up information, for at least 6 months. None of the patients had inflammatory diseases, trauma, abnormal lung or kidney function, rheumatic immune diseases, or hematological diseases for at least two weeks before IC or surgery. Furthermore, none of the patients had distant metastasis [12]. Among them, 62 patients received IC (IC group), and 66 patients immediately underwent a total laryngectomy after diagnosis (surgery group). In 62 IC patients, 39 preserved their laryngeal function (LFP) and formed the LFP group (2 chemotherapy after IC, 3 radiotherapy, 16 concurrent radiochemotherapy, and 18 partial laryngectomy). Nineteen patients underwent total laryngectomy and formed the TL group combined with other surgery group patients. Four patients preferred not to provide information about their treatments (Table 1 and Figure 1). There were 122 males and 6 females with a median age of 60 years old (range: 18–80). There were 89, 62, and 58 patients in T2/T3, N0, and stage III, respectively, and 39, 66, and 70 in T4, N1/N2/N3, and stage IV. In the IC group, 40 patients reached the treatment response for partial response (PR)/complete response (CR) and 22 for progressive disease (PD)/stable disease (SD). Patients’ pathological differentiation and primary lesions’ position are presented in Table 1.

### 2.2. Treatment Protocol

All treatment protocols have referred to NCCN Guidelines Version 1.2021, and all treatments have been evaluated and determined through our multidisciplinary team (MDT) discussion in Beijing Tongren Hospital Head and Neck Surgery after the diagnosis. The treatments were accepted by patients. Surgery group patients underwent a total laryngectomy by the same therapeutic group after diagnosis. IC group patients received two or three periodic induction chemotherapies of TPF (taxane/cisplatin/5-FU) at the beginning of diagnosis: Day 1 docetaxel 75 mg/m^2^ or paclitaxel 135 mg/m^2^, Days 2–4 cisplatin 30 mg/m^2^ per day, and Days 2–6 5-fluorouracil 500 mg/m^2^. The treatment was administrated every 3 weeks. After two periods, the IC response was evaluated by MDT according to a 2009 response evaluation criteria in solid tumors (RECIST) [13]: CR (disappearance of all target lesions with no more new lesions for at least four weeks), PR (a reduction in the sum of the target lesions’ largest diameters by more than 30%), PD (at least 20% increase in the sum of the target lesions’ largest diameters or new lesions appearance), and SD (patients between PD and PR) [14]. PR or CR responses were defined as sensitive (SEN), while PD or SD was defined as resistant (RES). In 62 IC group patients, those with response of SD, PD, or small PR (the tumor size decreased by less than 70%) underwent partial or total laryngectomy. Those with response of CR or large PR (the tumor size decreased by more than 70%) underwent radiotherapy, chemotherapy, or concurrent radiochemotherapy. Specific IC cycles, drug dosage, radiotherapy, and surgery methods were altered according to patients’ condition and preference (Figure 1).

### 2.3. Statistical Analysis

Peripheral blood parameters from blood routines, coagulation tests, and biochemical examinations were recorded at least two weeks before the initial treatment. Demographic information was collected, including age, gender, T stage, N stage, clinical stage, tumor position, pathological differentiation, and laryngeal function preservation. Overall survival (OS) was from the end of treatment to the death/the last time of follow-up (1 January 2022). Progression-free survival (PFS) was from the end of treatment to the progression of laryngeal carcinoma (the local recurrence or distant metastasis)/the last time of follow-up (1 January 2022). Disease-specific survival (DSS) was from the end of treatment to the death for laryngeal carcinoma/the last time of follow-up (1 January 2022). Statistical analysis was performed by SPSS software 24.0 (IBM Corporation, Armonk, NY, USA), GraphPad Prism 9 (San Diego, CA, USA), and R version 4.1.1 software. A Receiver Operating Characteristic (ROC) curve, the Kaplan–Meier (K-M) method, and a log-rank test were performed to screen predictive indicators for IC sensitivity and OS. The Cox proportional hazards model was used for multivariate survival analysis, and the indicators with a p-value less than 0.05 were involved in the model conduction. Logistic regression analysis and nomogram were performed to create the prediction model and calibration curve was used to evaluate the accuracy.

## 3. Results

### 3.1. The Study of Peripheral Blood Parameters to Predict IC Response in Advanced LSCC

#### 3.1.1. ROC Analysis to Select Potential Peripheral Blood Indicators

**Figure 2:**
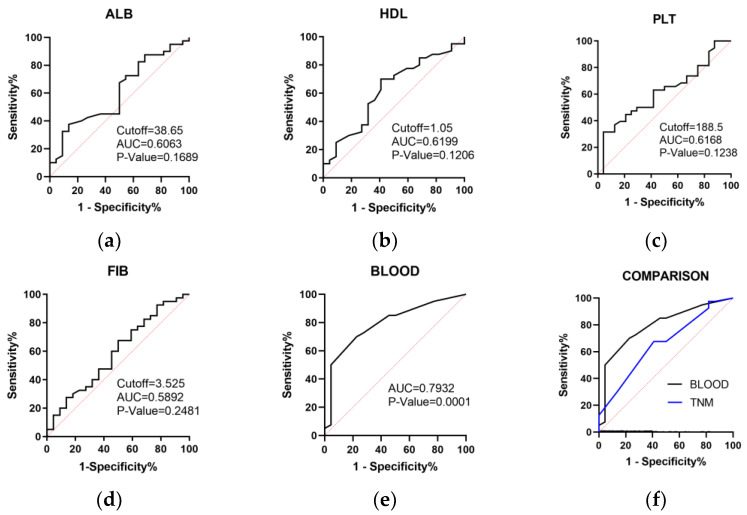
Receiver Operating Characteristic (ROC) analysis of peripheral blood parameters to predict induction chemotherapy (IC) response in advanced laryngeal squamous cell carcinoma (LSCC). (**a**–**d**) ROC analysis of peripheral blood parameters to predict IC response (AUC > 0.58); (**e**) ROC analysis of the blood model to predict IC response; (**f**) comparison between the blood model and conventional TNM stage model to predict IC response by ROC analysis.

ROC curves were performed to screen potential peripheral blood indicators and predict IC response, including platelet (PLT), neutrophilic granulocyte (NEUT), monocyte (MON), neutrophile to lymphocyte ratio (NLR), platelet to lymphocyte ratio (PLR), monocyte to lymphocyte ratio (MLR), hemoglobin (HGB), fibrinogen (FIB), lactate dehydrogenase (LDH), red blood cell distribution width (RDW), apolipoprotein-α (apoA-I), high-density lipoprotein cholesterol (HDL), albumin (ALB), plateletcrit (PCT), mean platelet volume (MPV), platelet distribution width (PDW), and systemic immune inflammation index (SII). Finally, four peripheral parameters were selected to predict IC response with an area under the curve (AUC) larger than 0.58, including FIB, PLT, HDL, and ALB (Figure 2a–d). The peripheral blood parameters were divided into high and low levels according to the cutoff values obtained by the Youden Index. The blood logistic model was constructed based on the four peripheral parameters for predicting IC response. The scores of the four indicators were defined as 1 value (≥cutoff) or 0 value (<cutoff). Then, ROC analysis was performed according to the predictor obtained by the logistic regression model and IC response sensitivity. Furthermore, the AUC of the blood logistic model reached 0.7932 by ROC analysis, which was significantly higher than the conventional TNM stage model (a logistic model conducted by the method mentioned above; the T, N, and M stages were divided into ranked variables according to the international criteria (T: 2, 3, 4; N: 0, 1, 2, 3; M: 0,1)) (Figure 2e,f).

#### 3.1.2. A Nomogram Model of Peripheral Blood Parameters Was Constructed to Predict IC Response

**Figure 3:**
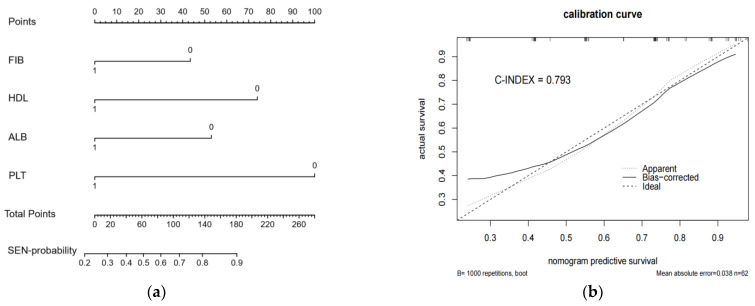
Nomogram (**a**) and calibration curve (**b**) of the blood model to predict IC response in advanced LSCC. The four blood indicators were categorical variables, where ≥ cutoff value is 1 and < cutoff value is 0.

To predict IC response effectively, we developed a nomogram consisting of the four selected peripheral blood parameters with a C-Index of 0.793 (Figure 3). In the nomogram, the four indicator scores were defined as 1 (≥cutoff) or 0 values (<cutoff), and the total scores were obtained by adding the individual scores of the peripheral blood parameters. Then, the response probability was calculated by the SEN-probability axis corresponding to the Total Points axis. The calibration curve showed good consistency between clinical observation and prediction.

### 3.2. The Study of Peripheral Blood Parameters to Predict Overall Survival in Advanced LSCC

#### 3.2.1. ROC and K-M Analysis to Select Potential Peripheral Blood Parameters to Predict OS

**Figure 4:**
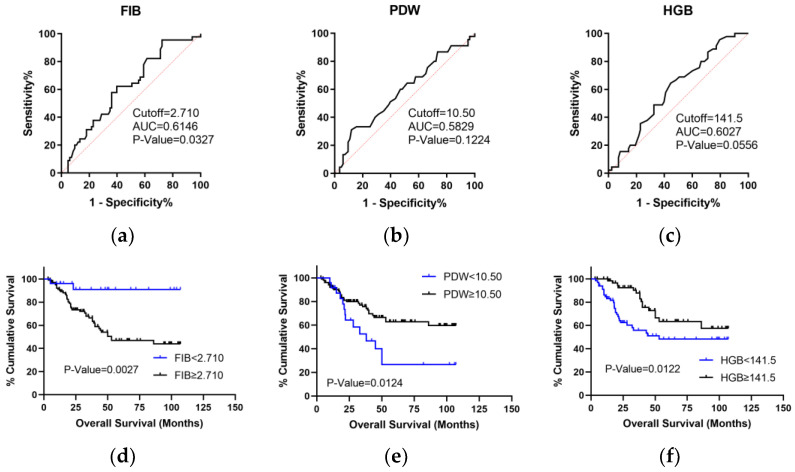
ROC and Kaplan–Meier (K-M) analyses of peripheral blood parameters to predict overall survival (OS) in LSCC. (**a**–**c**) ROC analysis of peripheral blood parameters to predict OS (AUC > 0.58). (**d**–**f**) K-M analysis of peripheral blood parameters for OS with the log-rank test (*p* < 0.05).

ROC curves were used to screen potential peripheral blood indicators for predicting OS, including PLT, NEUT, MON, NLR, PLR, MLR, HGB, FIB, LDH, RDW, apoA-Ⅰ, HDL, ALB, PCT, MPV, PDW, and SII. Thus, we selected three peripheral parameters with an AUC larger than 0.58 including FIB, PDW, and HGB. The peripheral parameters were divided into high and low levels according to the cutoff values obtained by the Youden Index. K-M analysis showed that patients’ OS with FIB < 2.710/PDW ≥ 10.500/HGB ≥ 141.500 was superior to patients with FIB ≥ 2.710/PDW < 10.500/HGB < 141.500 (*p* < 0.05, Figure 4).

#### 3.2.2. K-M Analysis to Select Clinical Factors Associated with OS

**Figure 5:**
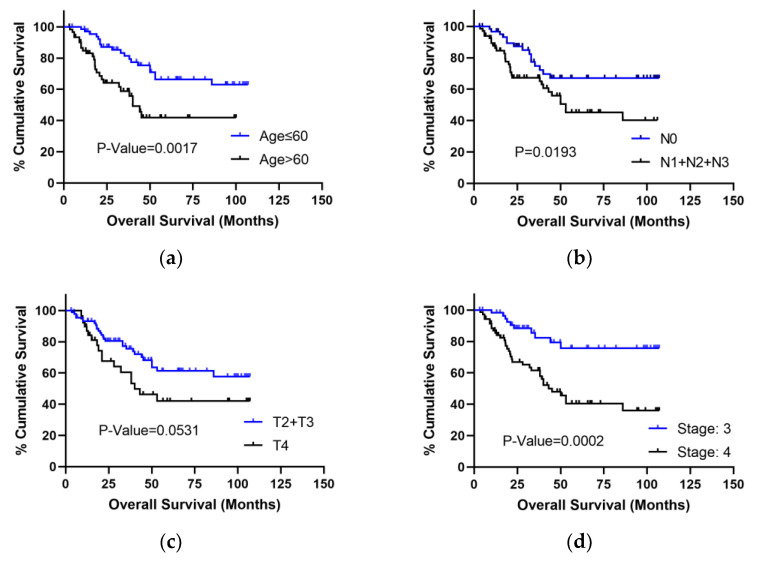
K-M analysis of demographic indicators to predict OS (*p*-value < 0.1) in LSCC. (**a**) K-M analysis of age for OS with the log-rank test. (**b**) K-M analysis of N stage for OS with the log-rank test. (**c**) K-M analysis of T stage for OS with the log-rank test. (**d**) K-M analysis of clinical stage for OS with the log-rank test.

The K-M analysis showed that the OS of patients aged less than 60 was superior to patients aged more than 60 by log-rank test (*p* < 0.05). The OS of patients without lymph node metastasis was superior to patients with lymph node metastasis (*p* < 0.05). The OS of patients with clinical stage III was superior to patients with stage IV by log-rank test (*p* < 0.05). There was no significant difference in OS between different T stages for advanced LSCC patients. However, there was a tendency for the OS of patients with T2 or T3 to be superior to that of patients with T4 (*p* = 0.0531) (Figure 5). In conclusion, age, lymph node metastasis, T stage, and clinical stage were selected for further study (*p* < 0.1).

#### 3.2.3. Multivariate COX Analysis to Select Independent Prognostic Factors for Advanced LSCC Patients

**Figure 6:**
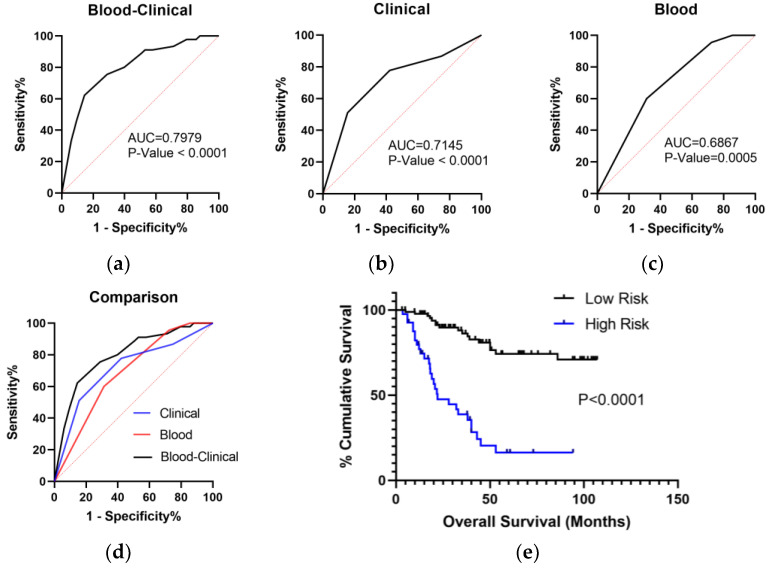
ROC analysis of the blood–clinical model to predict OS in LSCC. (**a**) ROC curve of the blood–clinical model involving fibrinogen (FIB), hemoglobin (HGB), age, and clinical stage. (**b**) ROC curve of the clinical model involving age and clinical stage. (**c**) ROC curve of the blood model involving FIB and HGB. (**d**) The comparison of AUC among the blood–clinical model, clinical model, and blood model. (**e**) According to the blood–clinical model, K-M analysis with a log-rank test of OS in patients with high or low risks.

The multivariate COX analysis included the selected peripheral blood parameters and clinical factors (Table 2). The scores of the three peripheral blood parameters were defined as 1 value (≥cutoff) or 0 value (<cutoff) according to the Youden Index, and the score of age was defined as 1 value (<60) or 0 value (≥60). T, N, and clinical stages were divided into ranked variables according to the international criteria. The results showed that age, stage, FIB, and HGB were independent prognostic factors for advanced LSCC patients (*p* < 0.05). Then, the blood–clinical logistic model was constructed to predict OS based on the four factors. ROC analysis showed that blood–clinical logistic model was superior to the blood and clinical models alone in predicting OS in advanced LSCC (AUC: 0.7979 vs. 0.6867 vs. 0.7145) (Figure 6a–d). The patients were divided into high-risk and low-risk by the blood–clinical logistic model. K-M analysis showed that low-risk patients’ OS was superior to the high-risk patients’ OS (Figure 6e).

#### 3.2.4. A Nomogram Model Was Constructed to Predict OS in LSCC Patients

**Figure 7:**
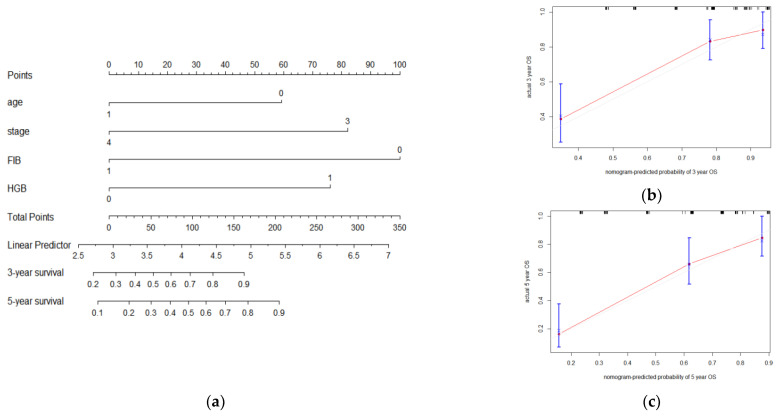
Nomogram analysis (**a**) and calibration (**b**,**c**) of the blood–clinical model for OS prediction in LSCC.

To predict OS, a nomogram was conducted involving the four independent prognosis factors based on multivariate COX analysis (*p* < 0.05), including age, stage, FIB, and HGB (C-Index = 0.792). In the nomogram, the scores of FIB and HGB were defined as 1 value (≥cutoff) or 0 value (<cutoff), and the score of age was defined as 1 (>60) or 0 values (≤60). The total score was obtained by adding the individual scores of the peripheral parameters and clinical indicators. Then, the three-year and five-year survival probabilities were calculated by the survival axis corresponding to the total-points axis. The calibration showed the good consistency between clinical observation and prediction (Figure 7).

### 3.3. K-M Analysis of OS, PFS, and DSS in Surgery Group and IC Group Patients

**Figure 8:**
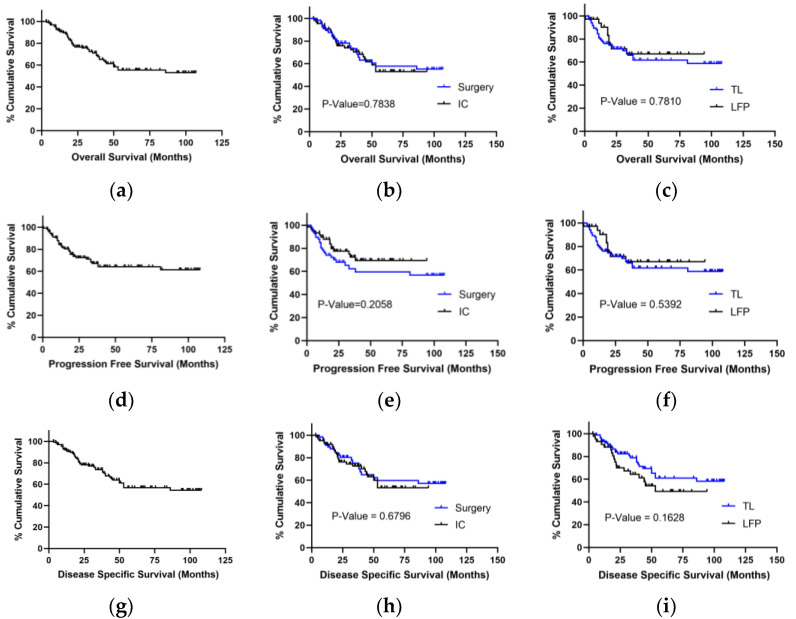
K-M analysis of OS, progression-free survival (PFS), and disease specific survival (DSS) in LSCC patients with a log-rank test. (**a**) K-M analysis of OS in all patients; (**b**,**c**) K-M analysis of treatment and laryngeal function preservation (LFP) or total laryngectomy (TL) for OS; (**d**) K-M analysis of PFS in all patients; (**e**,**f**) K-M analysis of treatment and LFP or TL for PFS; (**g**) K-M analysis of DSS in all patients; (**h**,**i**) K-M analysis of treatment and LFP or TL for DSS.

The OS, PFS, and DSS of all advanced LSCC patients were presented in Figure 8a,d,g, respectively. There were no significant differences in OS, PFS, and DSS between the IC and surgery groups by K-M analysis with the log-rank test. Among 62 IC group patients, 39 patients preserved laryngeal function (LFP group), and 19 patients received a total laryngectomy (TL). The LFP rate can reach up to 62.9%. Patients undergoing IC followed by TL and those surgery group patients were included in the TL group. There were still no significant differences in both OS, PFS, and DSS between the LFP and TL groups (Figure 8). Therefore, induction chemotherapy significantly improved laryngeal function preservation without lowering the survival prognosis.

## 4. Discussion

The response of advanced LSCC to IC shows heterogeneity. Some patients resistant to IC suffer adverse effects with no benefits [15]. Thus, it is urgent to find reliable indicators for predicting IC response prior to treatment. It has been confirmed that demographic and peripheral blood parameters are significantly correlated to prognosis in solid tumors [16]. However, it still lacks reliable models to predict IC response and OS by combining the two dimensions in advanced LSCC [17]. In our research, peripheral blood parameters were explored to predict IC response and overall survival in advanced LSCC. We constructed a blood logistic model based on the peripheral blood parameters (FIB, HDL, ALB, and PLT) to predict IC response (AUC = 0.7932) and a blood–clinical logistic model based on the four prognostic factors (age, stage, FIB, and HGB) to predict OS. Besides this, induction chemotherapy significantly improves the laryngeal function preservation without lowering the survival prognosis.

The overall survival and chemotherapy response of LSCC patients have been predicted by models consisting of histopathological factors (CA199, CA724), genes (CD48, CD2) [18], radiomics [19], and clinical factors (tumor differentiation, age, and stage) [20]. However, histopathological and gene factors have high testing costs and difficulty collecting samples. Radiomics and clinical factors are also less rigorous in some studies. An increasing number of studies have confirmed that peripheral blood parameters play an important role as biomarkers predicting prognosis and chemotherapy response in cancer since they are economically tested, and blood samples can be easily obtained [21].

Our study includes coagulation parameters of PLT and FIB in the blood model to predict IC response in LSCC. Tumor cells lead to coagulation status through neutrophil extracellular trap activation [22], increase PLT activation, and recruit platelets to bind with tumor tissue [23]. Moreover, platelets secrete transforming growth factor beta, plate factor 4, and lipoprotein-alpha to expedite tumor growth and epithelial-to-mesenchymal transition (EMT), in turn [24]. Some studies also found that platelets encapsulate circulating tumor cells and prevent them from being eliminated by natural killer (NK) cells [25]. Research about the expression of PLT in LSCC is still lacking. However, its predictive effect on prognosis has been confirmed in lung cancer, hepatocellular carcinoma, and so on [26]. FIB is also a prognostic factor for OS and disease-free survival, with a cutoff of 3.05 to 4.00 in LSCC [27]. Yanxy Han reports a significantly higher FIB in LSCC patients than in patients with benign laryngeal lesions [28]. The nutritional parameters of HGB, HDL, and ALB are involved in IC response and OS prediction models in our research. HGB reflects anemia and nutrition status; it has also been reported that anemia combined with a high NLR is significantly related to poor OS and PFS in LSCC [29]. Some pan-cancer research has indicated that tumor cells activate albumin metabolism and incur increasing albumin levels [30] despite other studies confirming that low expressions of HDL and ALB expressions are risk factors for patients’ survival [2]. Our research is the first to report the coagulation and nutritional parameters of FIB, PLT, HGB, HDL, and ALB, which may predict IC response and OS in LSCC. However, further studies are needed to explore their exact role and mechanism in laryngeal cancer.

The involvement of peripheral blood parameters improves prediction accuracy in OS and IC responses. In our study, age and clinical stage were independent prognostic factors for OS by multivariate COX analysis. The result aligns with the National Comprehensive Cancer Network (NCCN) guide, where the clinical stage is considered for patients’ stratification and treatment decisions [31]. We combined peripheral blood parameters with clinic factors to predict OS and improve prediction accuracy compared with the clinical model alone. Our study also shows that the blood model was superior to the TNM model for predicting IC response. These results imply that peripheral blood parameters are promising in IC response and OS prediction.

Surgery has always been the priority for advanced LSCC patients. Recently, laryngeal function preservation methods have been a concern. Francesco Bussu et al. reported that patients’ OS and DSS did not differ significantly between a laser endoscopic horizontal supraglottic laryngectomy group and an external approach group [32]. It has also been reported that there were no significant differences of prognosis between patients in the surgery and radiotherapy groups [33]. Patients with cricohyoidopexy showed a better LFP than radiochemotherapy [34]. IC has been used to extensively preserve organ function. A Veterans Affairs department has compared the efficiency of laryngeal function preservation and overall survival between different methods for laryngeal function preservation and found that induction chemotherapy followed by radiotherapy, as compared with radiotherapy alone, suppressed the development of distant metastases and improve disease-free survival [35]. Wolf G et al. also confirmed that IC followed by definitive radiotherapy in IC responders preserved the larynx function without lowering OS [36]. In our department, all treatment protocols have referred to NCCN Guidelines Version 1.2021, and all treatments have been evaluated and determined through our multidisciplinary team (MDT) discussion in Beijing Tongren Hospital Head and Neck surgery after the diagnosis [37]. As for T2N+ and T3 patients in our department, treatment modality (IC or partial laryngectomy or total laryngectomy) was chosen according to the tumor size, biopsy pathology, social factors, and the patients’ preference. As for T4 patients, although they were usually recommended a total laryngectomy, the tumor size, invaded border, involvement of vital vessel, patients’ condition, and larynx preservation preference were also considered; thus, some T4 patients underwent IC [32]. Our research confirmed no differences in OS, PFS, and DSS between LFP and TL groups and the IC and surgery groups. Among the 39 LFP patients, 15 died due to the disease progression (local recurrence, lymph node metastasis, or distant metastasis), except for one who died from primary lung cancer and another from hemorrhage. As for patients with TL, 26 died because of the disease progression, except for one surgery group patient who died from myocardial infarction. Induction chemotherapy significantly improved the laryngeal function preservation without lowering the survival prognosis. In addition, IC may not have an impact on mortality from other causes. Therefore, for some advanced LSCC patients sensitive to chemotherapy, IC is a priority treatment with benefits of preserving their laryngeal function.

There are some limitations in our retrospective and unicentral research. We lacked a validation set confirming the efficiency of the novel models. A larger sample and further research are needed. However, this remains a meaningful study with clinical advantages. Firstly, we created the blood model to predict IC response and the blood–clinical model to predict overall survival for LSCC. Both models have better efficiency than the conventional TNM model or single blood/clinical model. Secondly, all patients in our research were treated by the same therapeutic group, which ensured consistent treatment. Thirdly, we confirmed that IC increases LFP without lowering the OS, PFS, or DSS in our patients. Our research may help evaluate patients’ IC response, choose the more appropriate treatment modality between surgery and chemotherapy, and improve advanced LSCC patients’ quality of life.

## 5. Conclusions

In conclusion, we created effective models involving peripheral blood indexes that consider patients’ nutrition and coagulation. These models provide evidence for clinical decision making and prognosis evaluation. We also confirmed that IC improves laryngeal function preservation without reducing patients’ survival. Further studies with a larger sample and validation are needed.

## Figures and Tables

**Table 1:** Characteristics of laryngeal squamous cell carcinoma (LSCC) patients in the induction chemotherapy (IC) and surgery groups.

	IC	Surgery	Total	*p*-Value
**Gender**				1
Male	59	63	122	
Female	3	3	6	
**Age (years)**				0.607
>60	31	36	67	
≤60	31	30	61	
**T stage**				0.135
2 + 3	5 + 42	2 + 40	89	
4	15	24	39	
**N stage**				0.154
0	26	36	62	
1	3	17	20	
2	31	13	44	
3	2		2	
**M stage**				
0	62	66		
1	0	0		
**Differentiation**				0.706
High	15	12	27	
Moderate	35	40	75	
Poor	12	14	26	
**Position**				<0.05
Supraglottic	47	26	73	
Glottic + Subglottic/Others	13 + 2	36 + 4	55	
**LFP OR TL**				
LFP ^1^ (radio + chemo + CRT ^7^ + PL ^8^)	3 + 2 + 16 + 18	0	39	
TL ^2^	19	66	85	
**Clinical Stage**				0.272
3	25	33	58	
4	37	33	70	
**Response to IC**				
PD ^3^ + SD ^4^	22		22	
CR ^5^ + PR ^6^	40		40	

^1^: laryngeal function preservation; ^2^: total laryngectomy; ^3^: progressive disease; ^4^: stable disease; ^5^: complete response; ^6^: partial response; ^7^: concurrent radiochemotherapy; ^8^: partial laryngectomy.

**Table 2:** Multivariate COX regression of demographic factors and peripheral parameters for OS in all advanced LSCC patients.

Variate	B	Exp(B)	*p*-Value
Age	0.869	2.384 (1.272–4.466)	0.007 *
T	−0.200	0.818 (0.444–1.508)	0.520
N	−0.042	0.958 (0.650–1.413)	0.830
Stage	1.581	4.861 (1.759–13.427)	0.002 *
FIB	1.867	6.470 (1.522–27.500)	0.011 *
HGB	−1.215	0.297 (0.153–0.577)	<0.001 *
PDW	−0.641	0.527 (0.256–1.083)	0.081

* The significant prognostic factors with *p*-value < 0.05.

## Data Availability

The data presented in this study are available on request from the corresponding author.
